# Supplementary material for: Cinnamic Acid Attenuates Peripheral and Hypothalamic Inflammation in High-Fat Diet-Induced Obese Mice
Source: Pharmaceutics. 2022 Aug 11;14(8):1675. doi: 10.3390/pharmaceutics14081675 (PMC9413375; doi:10.3390/pharmaceutics14081675)
Supplement: Supplementary file 1 [file pharmaceutics-14-01675-s001.zip › pharmaceutics-1815053-supplementary.pdf]

Supplementary Table S1. A list of 82 active compounds in the Cinnamon

Compound	Molecular Formula	Molecular Weight (g/mol)
(3-ethoxy-hexa-1,5-dienyl)-benzene	$C_{14}H_{18}O$	202.29
1-heptadecene	$C_{17}H_{34}$	238.45
1,8-cineole	$C_{10}H_{18}O$	154.25
3-methylbenzothiophene	C_9H_8S	148.22
4-(2-propenyl)-phenol	$C_9H_{10}O$	134.18
5-(2-propenyl)-1,3-benzodioxole	$C_{10}H_{10}O_2$	162.19
6-Glucopyranosylprocyanidin B1	$C_{36}H_{36}O_{17}$	740.7
aromadendrene	$C_{15}H_{24}$	204.35
benzaldehyde	C_7H_6O	106.12
benzeneethanol	$C_8H_{10}O$	122.16
benzenemethanol	C_7H_8O	108.14
benzenepropanol	$C_9H_{12}O$	136.19
benzyl benzoate	$C_{14}H_{12}O_2$	212.24
bicyclogermacrene	$C_{15}H_{24}$	204.35
borneol	$C_{10}H_{18}O$	154.25
caffeic acid	$C_9H_8O_4$	180.16
camphor	$C_{10}H_{16}O$	152.23
caryophyllene	$C_{15}H_{24}$	204.35
catechin	$C_{15}H_{14}O_6$	290.27
chlorogenic acid	$C_{16}H_{18}O_9$	354.31
cinnamaldhyde	C_9H_8O	132.16
cinnamic acid	$C_9H_8O_2$	148.16
cinnamtannin-B1	$C_{45}H_{36}O_{18}$	864.76
cinnamyl acetate	$C_{11}H_{12}O_2$	176.21
cinnamyl alcohol	$C_9H_{10}O$	134.18
cis-isoeugenol	$C_{10}H_{12}O_2$	164.2
copaene	$C_{15}H_{24}$	204.35
coumarin	$C_9H_6O_2$	146.14
cubenol	$C_{15}H_{26}O$	222.37
cymene	$C_{10}H_{14}$	134.22
docosane	$C_{22}H_{46}$	310.6
epicatechin	$C_{15}H_{14}O_6$	290.27
eugenol	$C_{10}H_{12}O_2$	164.2
eugenol acetate	$C_{12}H_{14}O_3$	206.24
ferulic acid	$C_{10}H_{10}O_4$	194.18
gallic acid	$C_7H_6O_5$	170.12
germacrene-d	$C_{15}H_{24}$	204.35
hydrocinnamaldehyde	$C_9H_{10}O$	134.18
isospathulenol	$C_{15}H_{24}O$	220.35
kaempferol 3-alpha-L-arabinofuranoside-7-rhamnoside	$C_{26}H_{28}O_{14}$	564.5
limonene	$C_{10}H_{16}$	136.23
linalool	$C_{10}H_{18}O$	154.25
methoxy-eugenol	$C_{11}H_{14}O_3$	194.23
neohexane	C_6H_{14}	86.18

neophytadiene	$C_{20}H_{38}$	278.52
nonadecene	$C_{19}H_{38}$	266.51
octacosane	$C_{28}H_{58}$	394.76
ortho-methoxy cinnamaldehyde	$C_{10}H_{10}O_2$	162.18
p-coumaric acid	$C_9H_8O_3$	164.16
p-cymene	$C_{10}H_{14}$	134.22
p-hydroxybenzoic acid	$C_7H_6O_3$	138.12
p-vinylbenzohydrazide	$C_9H_{10}N_2O$	162.19
pavetannin-C1	$C_{60}H_{48}O_{24}$	1153
proanthocyanidin-A2	$C_{30}H_{24}O_{12}$	576.5
procyanidin-B7	$C_{30}H_{26}O_{12}$	578.5
protocatechuic acid	$C_7H_6O_4$	154.12
quercetin	$C_{15}H_{10}O_7$	302.24
rosmarinic acid	$C_{18}H_{16}O_8$	360.31
safrole	$C_{10}H_{10}O_2$	162.19
salicylic acid	$C_7H_6O_3$	138.12
sinapic acid	$C_{11}H_{12}O_5$	224.21
spathulenol	$C_{15}H_{24}O$	220.35
syringic acid	$C_9H_{10}O_5$	198.17
tenuifolin	$C_{36}H_{56}O_{12}$	680.8
terpinen-4-ol	$C_{10}H_{18}O$	154.25
tetracosane	$C_{24}H_{50}$	338.65
trans-cinnamaldehyde	C_9H_8O	132.16
vanillic acid	$C_8H_8O_4$	168.15
vanillin	$C_8H_8O_3$	152.15
viridiflorol	$C_{15}H_{26}O$	222.37
α-amorphene	$C_{15}H_{24}$	204.35
α-caryophyllene	$C_{15}H_{24}$	204.35
α-copaene	$C_{15}H_{24}$	204.35
α-muurolene	$C_{15}H_{24}$	204.35
α-phellandrene	$C_{10}H_{16}$	136.23
α-Pinene	$C_{10}H_{16}$	136.23
α-terpineol	$C_{10}H_{18}O$	154.25
α-thujene	$C_{10}H_{16}$	136.23
β-bisabolene	$C_{15}H_{24}$	204.35
β-caryophyllene	$C_{15}H_{24}$	204.35
β-terpinene	$C_{10}H_{16}$	136.23
δ-elemene	$C_{15}H_{24}$	204.35

Supplementary Table S2. Number of violations of cinnamon compounds for "drug likeness" via different pharmacokinetic filters

Compound	Filters[a] for drug likeness					
	Lipinski	Ghose	Veber	Egan	Muegge	Bioavailability Score
Cinnamic acid	0	2	0	0	1	0.85
Ferulic acid	0	0	0	0	1	0.85
p-Coumaric acid	0	0	0	0	1	0.85
p-Hydroxybenzoic acid	0	3	0	0	1	0.85
Salicylic acid	0	3	0	0	1	0.85

[a] Lipinski's filter: MW ≤ 500 Da, number of H-bond donors ≤ 5, number of H-bond acceptors ≤ 10, logarithm of octanol-water partition coefficient (logP) ≤ 5;
Ghose's filter: 160 ≤ MW ≤ 480, −0.4 ≤ WLOGP (lipophilicity) ≤ 5.6, 40 ≤ the molar refractivity ≤ 130, 20 ≤ number of atoms ≤ 70;
Veber's filter: the number of rotatable bonds ≤ 10, the total polar surface area ≤ 140;
Egan's filter: WLOGP (lipophilicity) ≤ 5.88, the total polar surface area ≤ 131;
Muegge's filter, 200 ≤ MW ≤ 600, −2 ≤ XLOGP3 (lipophilicity) ≤ 5, the total polar surface area ≤ 150, the number of rings ≤ 7, the number of carbons > 4, the number of heteroatoms > 1, the number of rotatable bonds ≤15, number of H-bond acceptors ≤ 10, number of H-bond donors ≤ 5.

Supplementary Table S3. Physicochemical properties of cinnamon compounds

Compound	Physicochemical Properties									
	Formula	MW	#Heavy atoms	#Aromatic heavy atoms	Fraction Csp3	#Rotatable bonds	#H-bond acceptors	#H-bond donors	MR	TPSA
Cinnamic acid	$C_9H_8O_2$	148.16	11	6	0	2	2	1	43.11	37.3
ferulic acid	$C_{10}H_{10}O_4$	194.18	14	6	0.1	3	4	2	51.63	66.76
p-coumaric acid	$C_9H_8O_3$	164.16	12	6	0	2	3	2	45.13	57.53
p-hydroxybenzoic acid	$C_7H_6O_3$	138.12	10	6	0	1	3	2	35.42	57.53
salicylic acid	$C_7H_6O_3$	138.12	10	6	0	1	3	2	35.42	57.53

MW, molecular weight; Fraction Csp3, ratio of sp3 hybridized carbons over the total carbon count of the molecule; MR, molecular refractivity; TPSA, topological polar surface area.

Supplementary Table S4. Lipophilicity of cinnamon compounds

Compound	Lipophilicity					
	iLOGP	XLOGP3	WLOGP	MLOGP	Silicos-IT Log P	Consensus Log P
Cinnamic acid	1.55	2.13	1.68	1.9	1.7	1.79
Ferulic acid	1.62	1.51	1.39	1	1.26	1.36
p-Coumaric acid	0.95	1.46	1.38	1.28	1.22	1.26
p-Hydroxybenzoic acid	0.85	1.58	1.09	0.99	0.74	1.05
Salicylic acid	1.13	2.26	1.09	0.99	0.74	1.24

iLOGP, implicit log *P* is an in-house physics-based methods relying on Gibbs free energy of solvation calculated by GB/SA in water and *n*-octanol; XLOGP3, the logP value of a query compound by using the known logP value of a reference compound as a starting point; WLOGP, log P method developed by Wildman and Crippen; MLOGP, Moriguchi octanol-water partition coefficient; SILICOS-IT, an hybrid method relying on 27 fragments and 7 topological descriptors; Consensus Log P, arithmetic mean of the values predicted by the five proposed methods.

Supplementary Table S5. Water solubility of cinnamon compounds

Compound	Water Solubility								
	ESOL Solubility			Ali Solubility			Silicos-IT Solubility		
	Log S	(mg/ml)	(mol/l)	Log S	(mg/ ml)	(mol/l)	LogSw	(mg/ml)	(mol/l)
Cinnamic acid	-2.37	-2.37	0.629	-2.54	0.423	0.002850	-1.84	2.14	0.0145
Ferulic acid	-2.16	-2.11	1.490	-2.52	0.586	0.003020	-1.42	7.43	0.0383
p-Coumaric acid	-2.17	-2.02	1.580	-2.27	0.873	0.005320	-1.28	8.67	0.0528
p-Hydroxybenzoic acid	-2.43	-2.07	1.180	-2.4	0.551	0.003990	-1.17	9.40	0.0681
Salicylic acid	-2.46	-2.5	0.439	-3.1	0.109	0.000786	-1.17	9.40	0.0681

ESOL, Estimating Aqueous Solubility Directly from Molecular Structure; Ali, *in silico* prediction model of aqueous solubility revised by Ali J.; Silicos-IT, predictor for solubility developed by SILICOS-IT; LogSw, decimal logarithm of the molar solubility in water.

Supplementary Table S6. Pharmacokinetics of cinnamon compounds

Compound	Pharmacokinetics								
	GI	BBB	Pgp	CYP inhibition					log Kp (cm/s)
				1A2	2C19 i	2C9	2D6	3A4	
Cinnamic acid	-4.388	0.961	0.059	0.082	0.036	0.021	0.117	0.001	-5.69
Ferulic acid	-4.943	0.565	0.026	0.234	0.061	0.059	0.184	0.012	-6.41
p-Coumaric acid	-4.884	0.451	0.036	0.054	0.035	0.087	0.218	0.011	-6.26
p-Hydroxybenzoic acid	-4.844	0.18	0.019	0.023	0.006	0.007	0.111	0.001	-6.02
salicylic acid	-4.851	0.379	0.027	0.003	0.013	0.007	0.103	0.001	-5.54

GI, gastrointestinal absorption; BBB, blood-brain barrier permeant; Pgp, permeability glycoprotein substrate; CYP, cytochrome P450; log Kp, decimal logarithm of the skin permeability coefficient

Supplementary Table S7. Molecular docking binding affinity of cinnamon components to TNF-α, MIF from M1 macrophage, and MMP-12 from M2 macrophage.

Compound	Binding Affinity (kcal/mol)		
	TNF-α	MIF	MMP-12
Cinnamic acid	-5.9	-7.2	-6.6
Ferulic acid	-6.6	-6.6	-6.2
p-Coumaric acid	-6.2	-7.1	-7.0
p-Hydroxybenzoic acid	-5.3	-6.4	-6.2
Salicylic acid	-5.4	-6.6	-6.4

Tumor necrosis factor-α (TNF-α) (PDBID: 2AZ5) as an inflammatory cytokine, MIF (PDBID: 1GD0) as a pro-inflammatory macrophage, MMP-12 (PDBID: 1JIZ) as an anti-inflammatory macrophage.

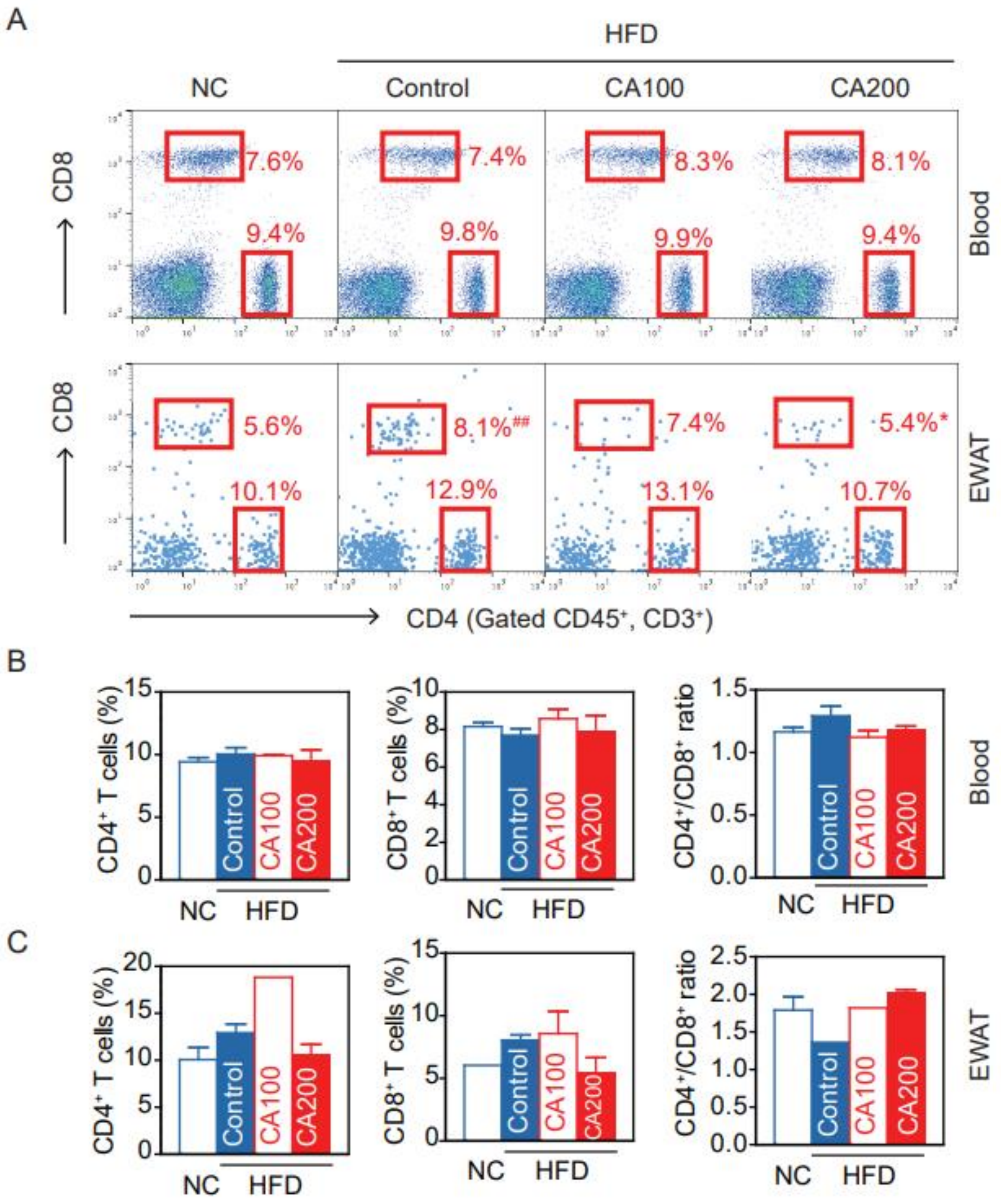

Supplementary Figure S1. T cell subtype in blood and epididymal white adipose tissue (EWAT) of HFD-induced obesity. (A) Flow cytometry analyses showing the fractions of T cells in blood or EWAT. (B) percentage of blood $CD4^+$ T cells, percentage of blood $CD8^+$ T cells, and ratio of $CD4^+$ and $CD8^+$ T cell in blood, (C) percentage of EWAT $CD4^+$ T cells, percentage of blood $CD8^+$ T cells, and ratio of $CD4^+$ and $CD8^+$ T cell in EWAT. Data are expressed as the mean ± SEM. NC: normal chow, Control: high-fat diet control, CA100: high-fat diet plus cinnamic acid 100 mg/kg, CA200: high-fat diet plus cinnamic acid 200 mg/kg.
